# Supplementary material for: Quality Evaluation Based Simulation Selection (QEBSS) for analysis of conformational ensembles and dynamics of multidomain proteins
Source: Commun Chem. 2025 Aug 10;8:241. doi: 10.1038/s42004-025-01623-x (PMC12335556; doi:10.1038/s42004-025-01623-x)
Supplement: Supplementary file 2 — Supplemental Information [file 42004_2025_1623_MOESM2_ESM.pdf]

Supplementary material: Quality Evaluation Based Simulation Selection (QEBSS) for analysis of conformational ensembles and dynamics of multi-domain proteins

Amanda E. Sandelin,[†,‡] Ricky Nencini,[¶,‡] Ekrem Yasar,[§] Satoshi Fudo,[‡] Vassilis Stratoulias,[†] Tommi Kajander,[‡] and O. H. Samuli Ollila[*,∥,‡]

†*Division of Pharmacology and Pharmacotherapy, Faculty of Pharmacy, University of Helsinki, Helsinki, Finland*

‡*Institute of Biotechnology, University of Helsinki, 00014 Helsinki, Finland*

¶*Division of Pharmaceutical Biosciences, Faculty of Pharmacy, University of Helsinki, Helsinki, Finland*

§*Department of Biophysics, Faculty of Medicine, Erzincan Binali Yildirim University, 24100, Erzincan, Turkey*

∥*VTT Technical Research Centre of Finland, 02044 Espoo, Finland*

E-mail: samuli.ollila@vtt.fi

Supplementary results

Radius of gyration distributions

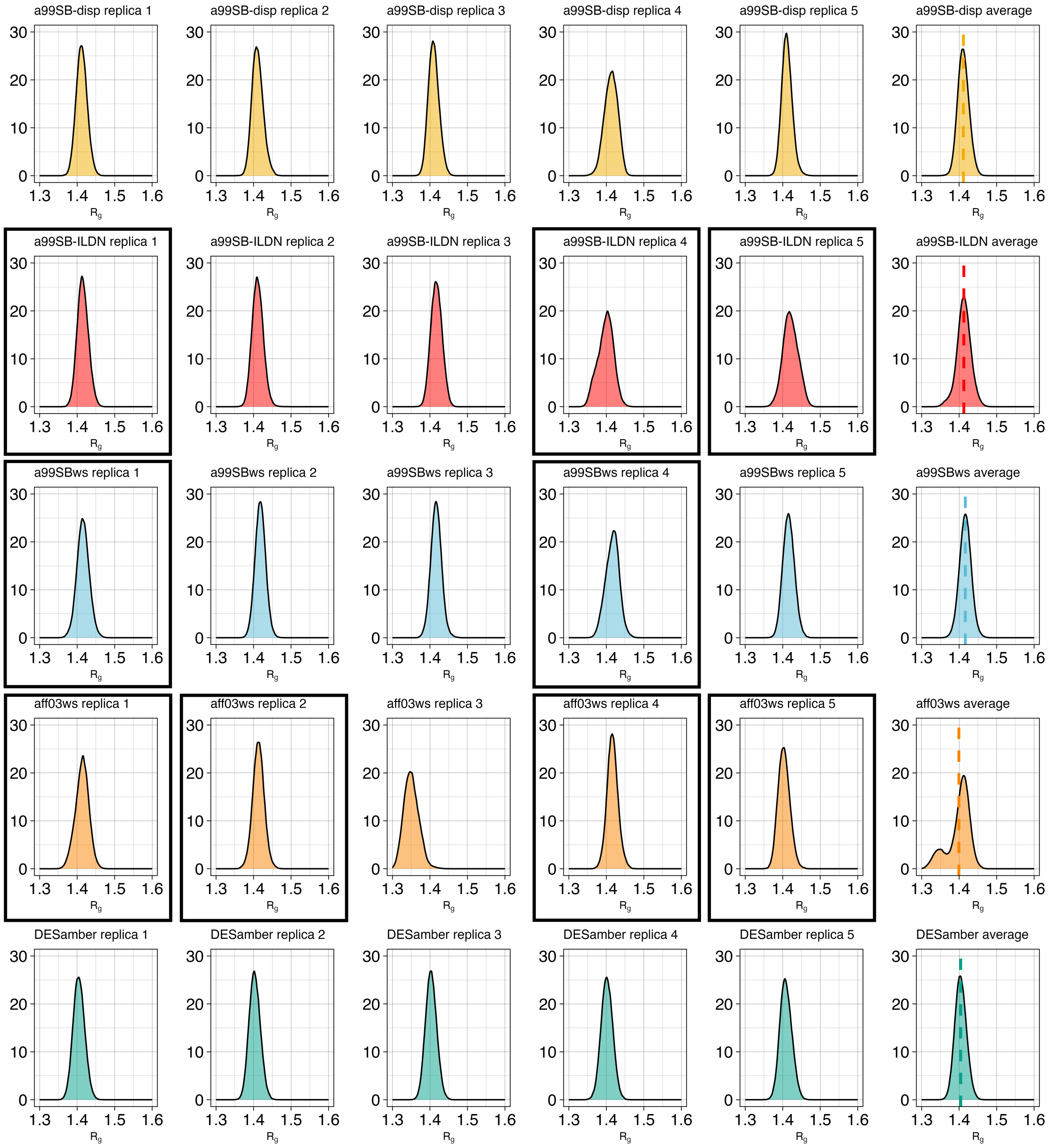

Figure S1: Radius of gyration (R_g in nm) distributions from all MD simulation replicas and force field separately, and averages over replicas (rightmost column) for TonBCTD. The dashed line represents the average radius of gyration. QEBSS-selected replicas are highlighted with a black box.

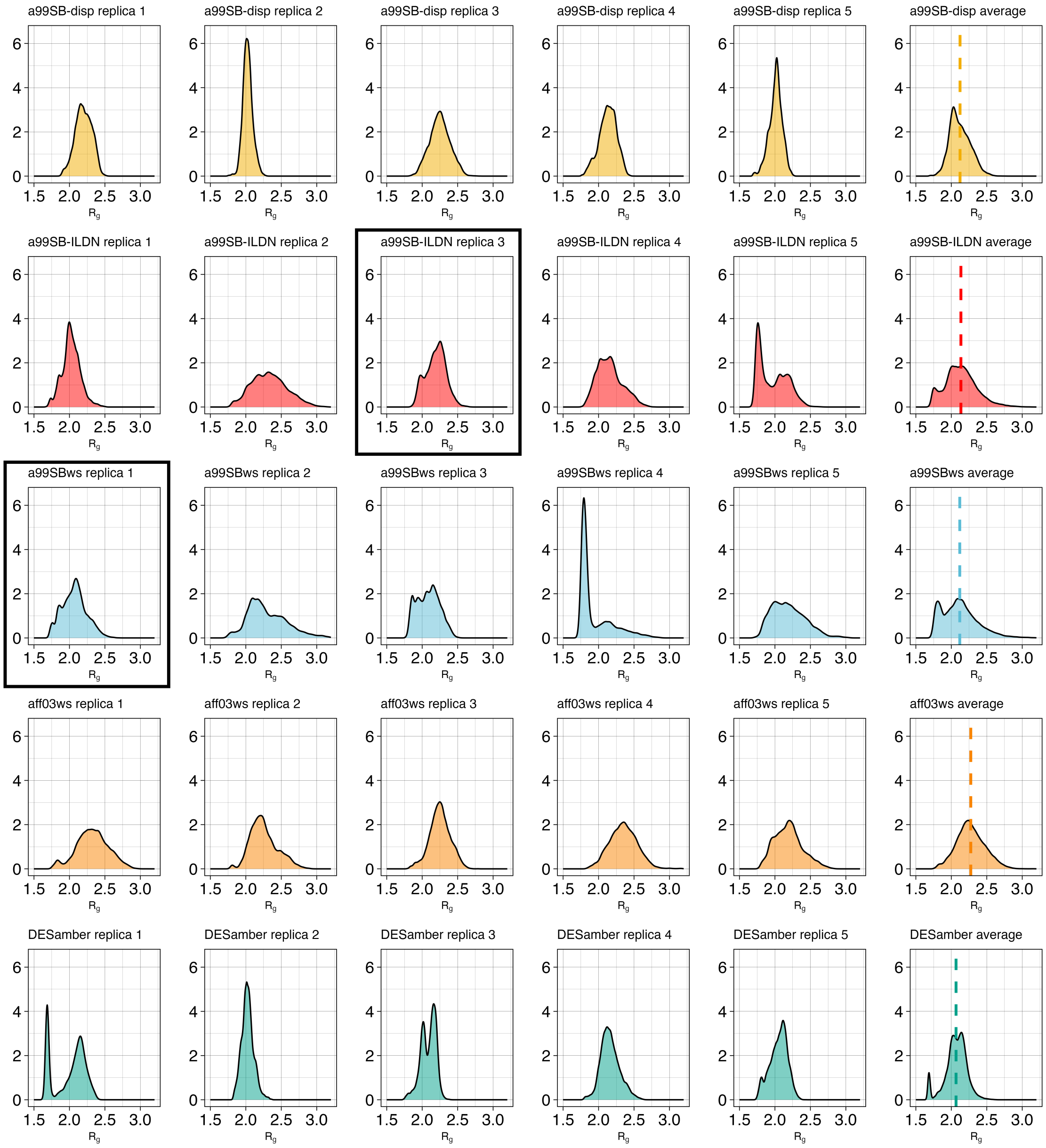

Figure S2: Radius of gyration (R_g in nm) distributions from all MD simulation replicas and force field separately, and averages over replicas (rightmost column) for calmodulin. The dashed line represents the average radius of gyration. QEBSS-selected replicas are highlighted with a black box.

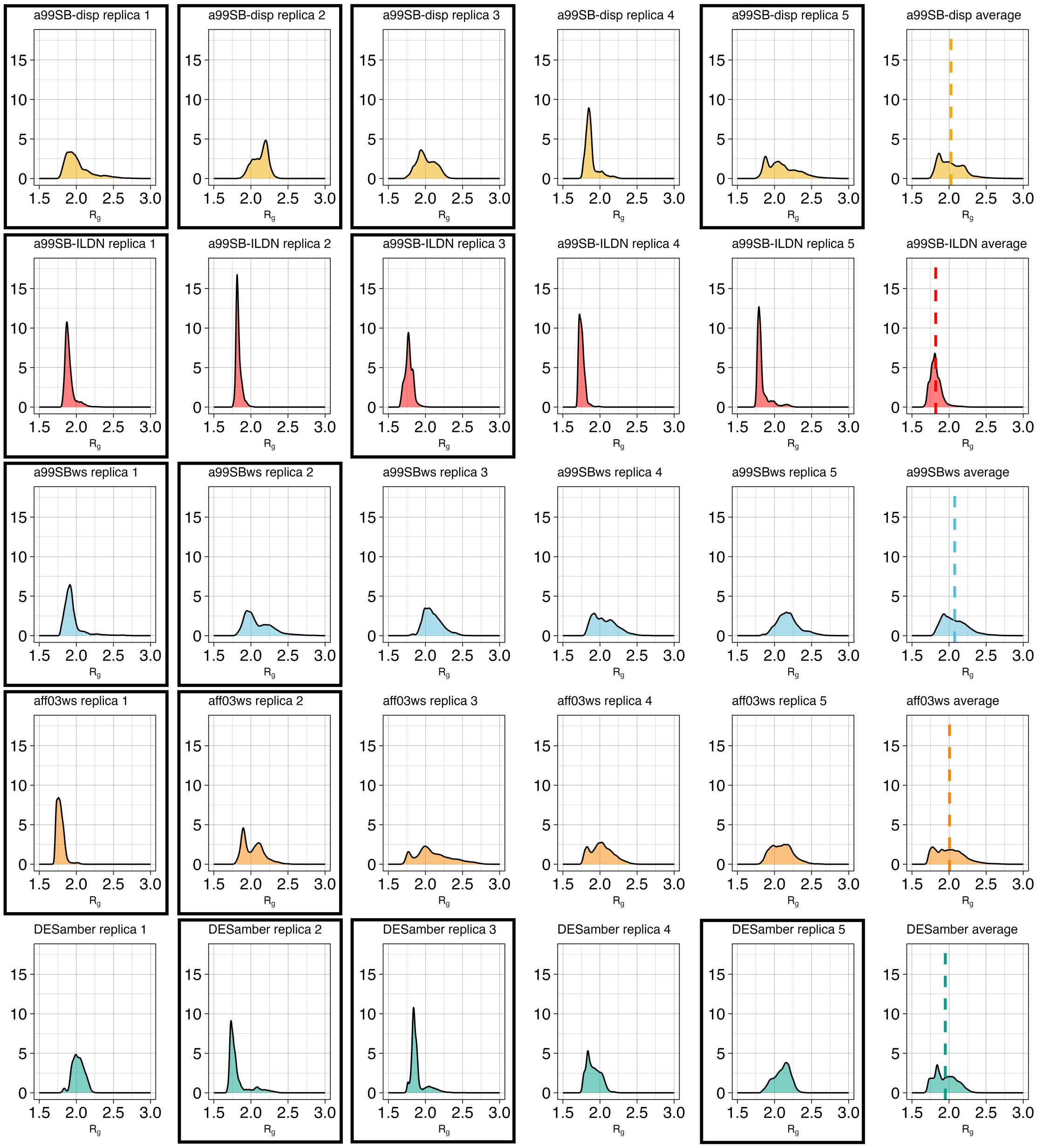

Figure S3: Radius of gyration (R_g in nm) distributions from all MD simulation replicas and force field separately, and averages over replicas (rightmost column) for CDNF. The dashed line represents the average radius of gyration. QEBSS-selected replicas are highlighted with a black box.

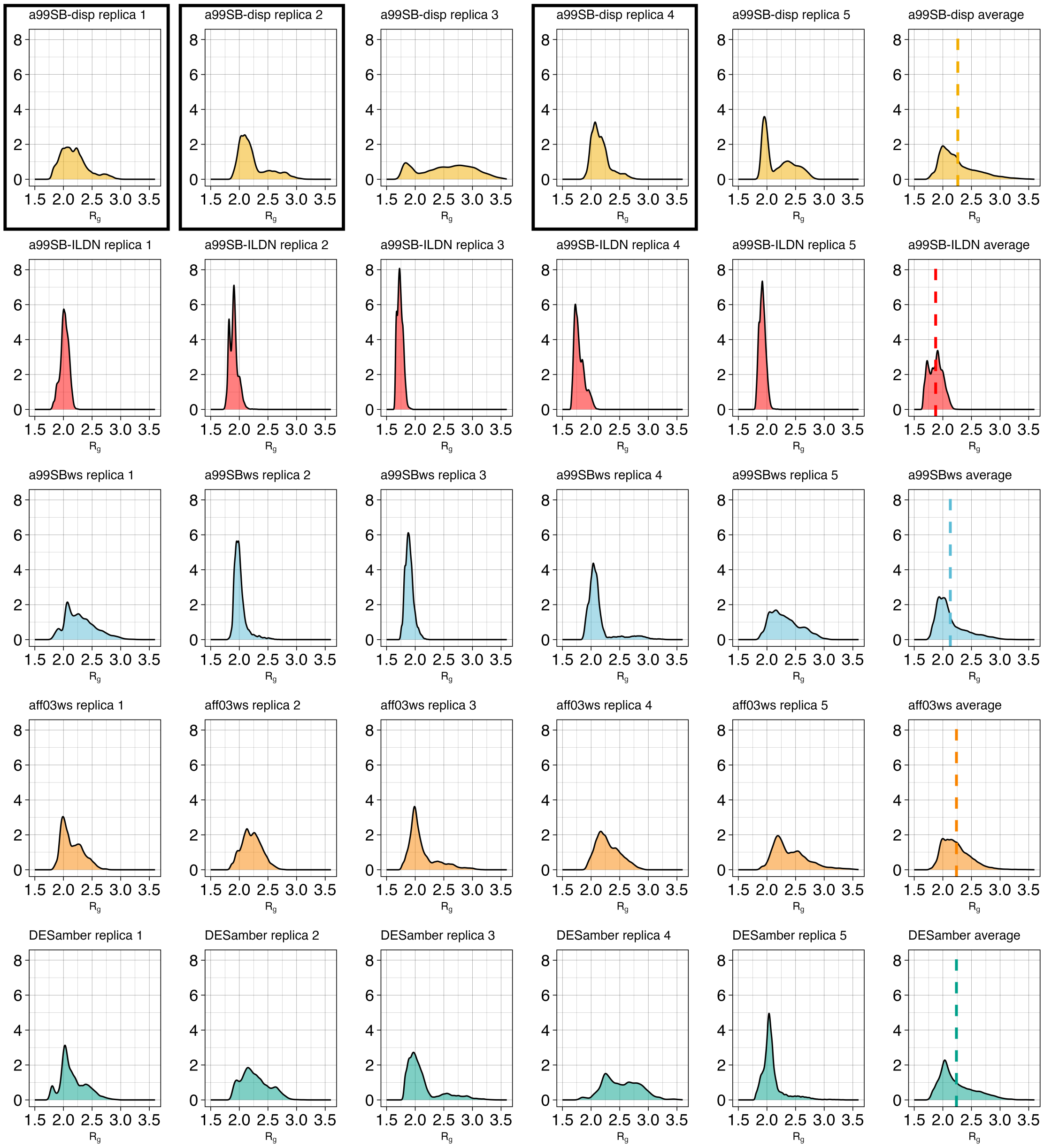

Figure S4: Radius of gyration (R_g in nm) distributions from all MD simulation replicas and force field separately, and averages over replicas (rightmost column) for MANF. The dashed line represents the average radius of gyration. QEBSS-selected replicas are highlighted with a black box.

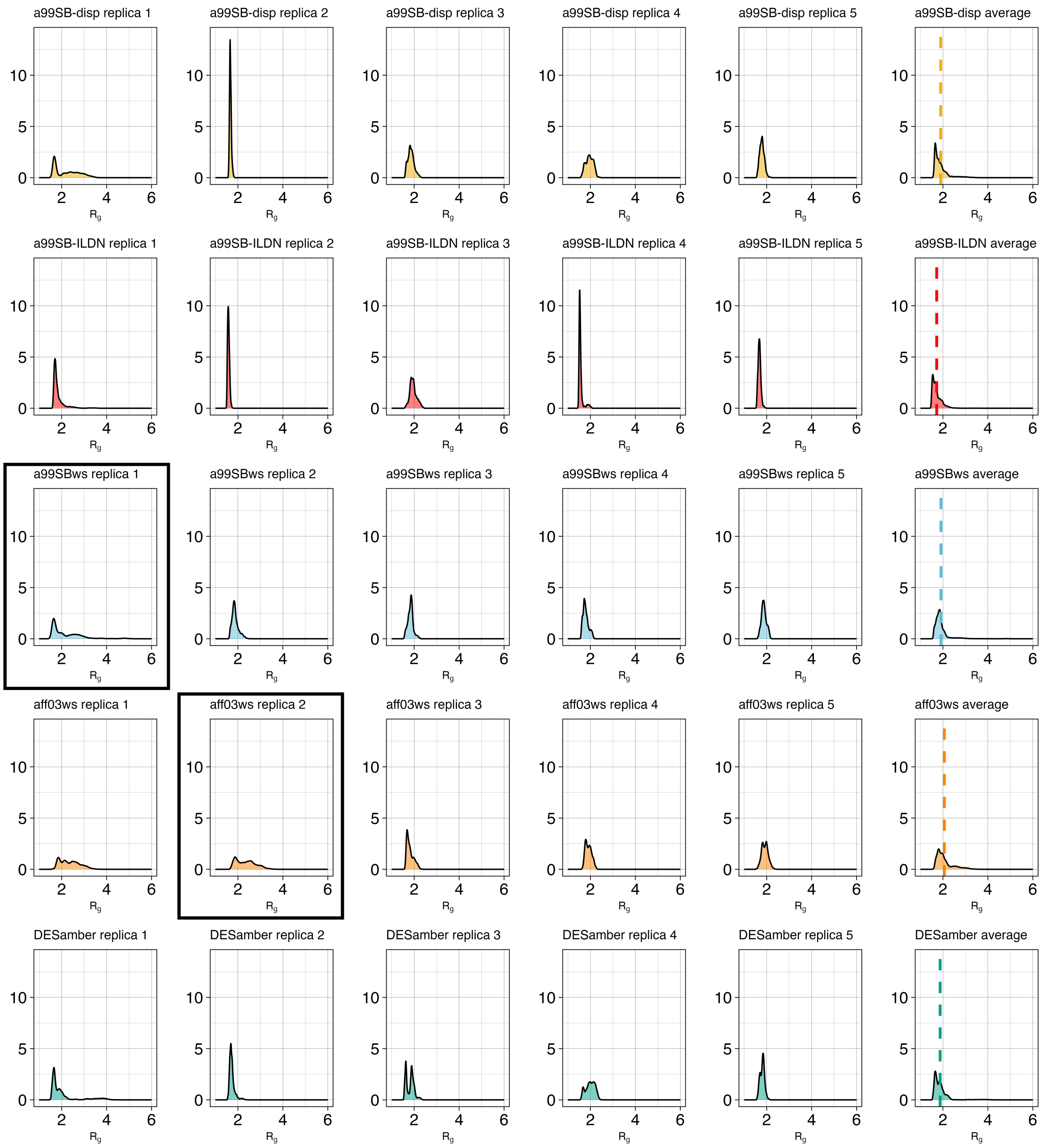

Figure S5: Radius of gyration (R_g in nm) distributions from all MD simulation replicas and force field separately, and averages over replicas (rightmost column) for EN2. The dashed line represents the average radius of gyration. QEBSS-selected replicas are highlighted with a black box.

Backbone orientation correlation

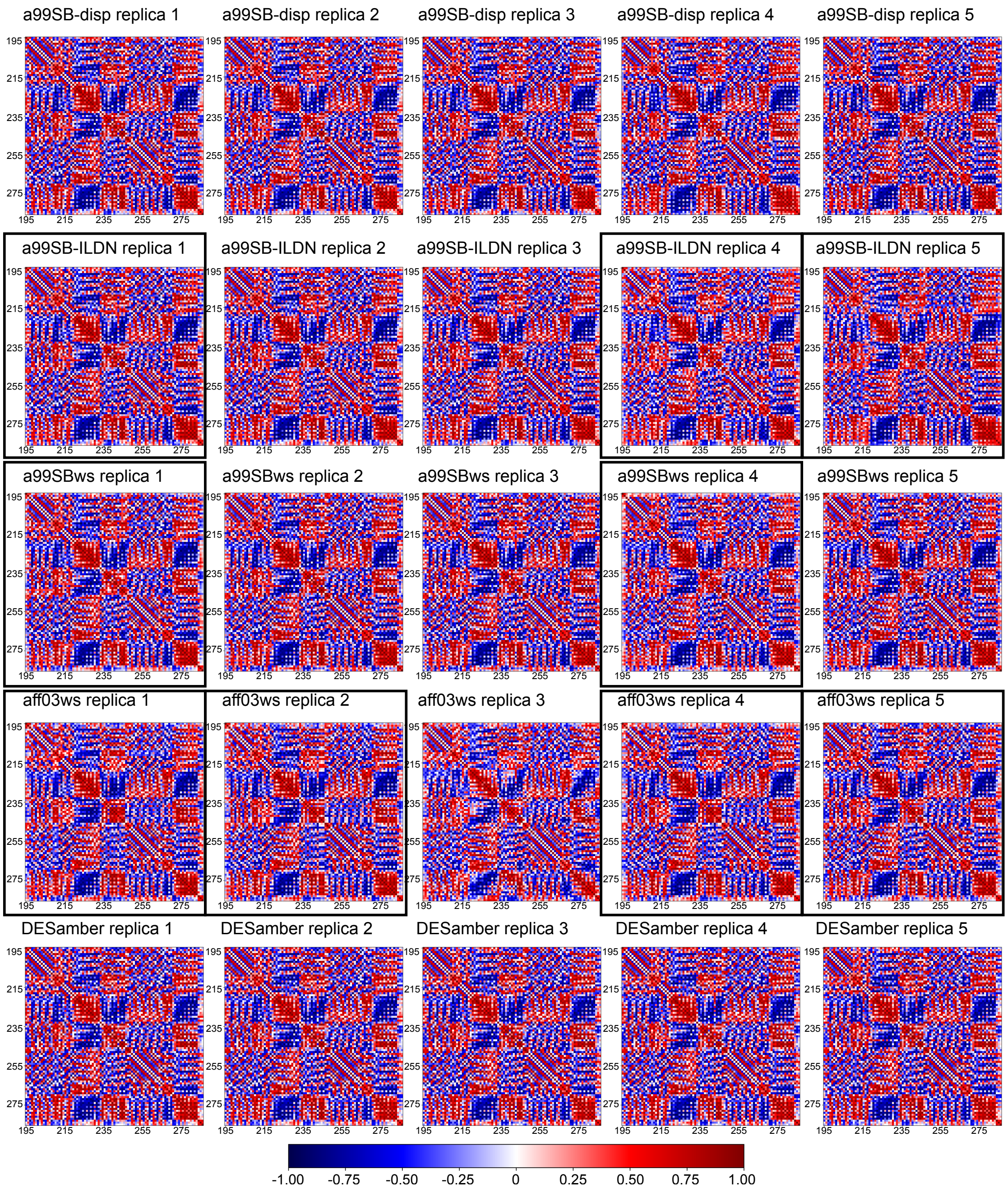

Figure S6: Protein backbone orientation correlation maps for vectors between C^{α} carbons of consecutive residues of all molecular dynamics (MD) simulation replicas for TonBCTD. QEBSS-selected replicas are highlighted with a black box.

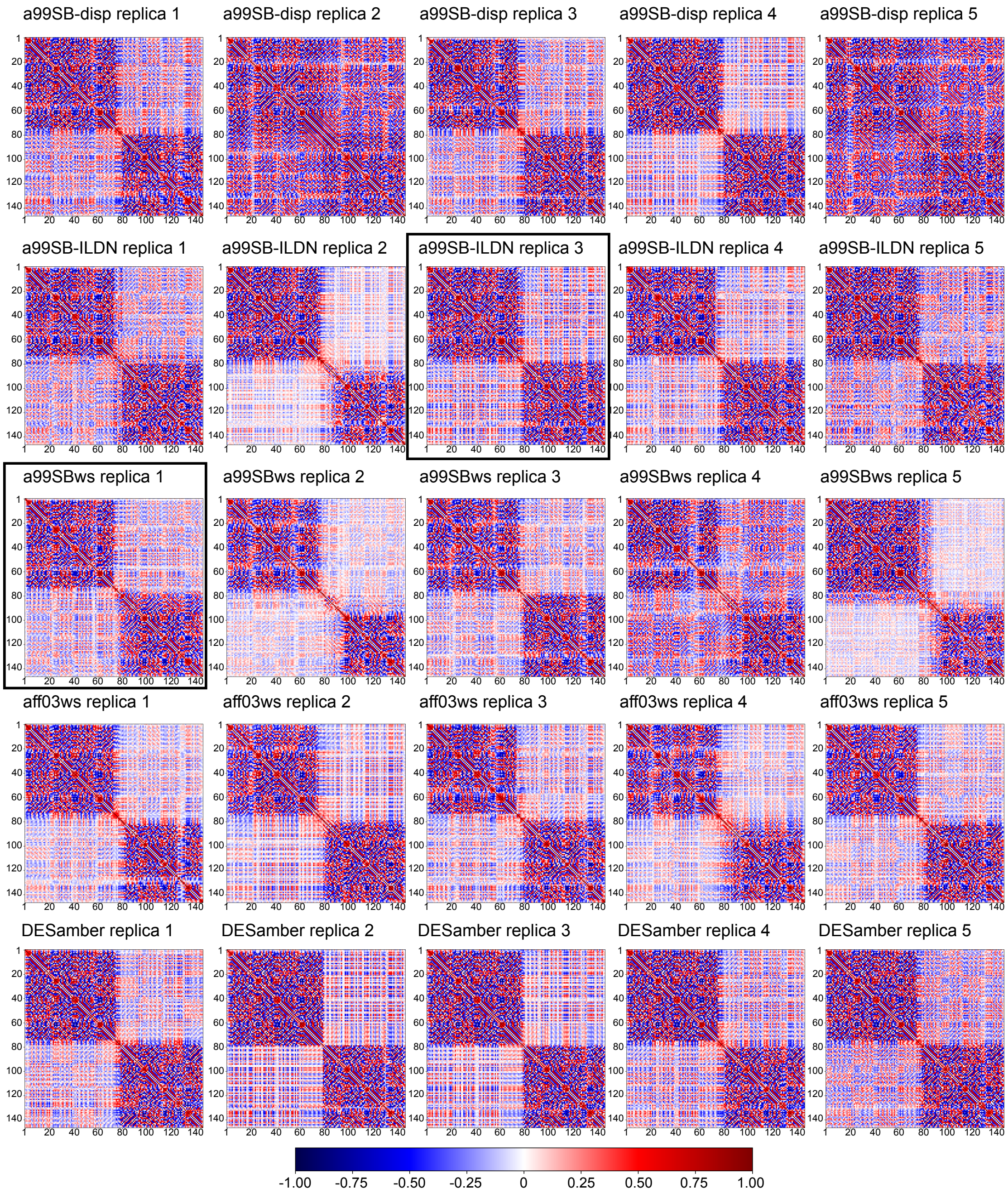

Figure S7: Protein backbone orientation correlation maps for vectors between C^{α} carbons of consecutive residues of all molecular dynamics (MD) simulation replicas for calmodulin. QEBSS-selected replicas are highlighted with a black box.

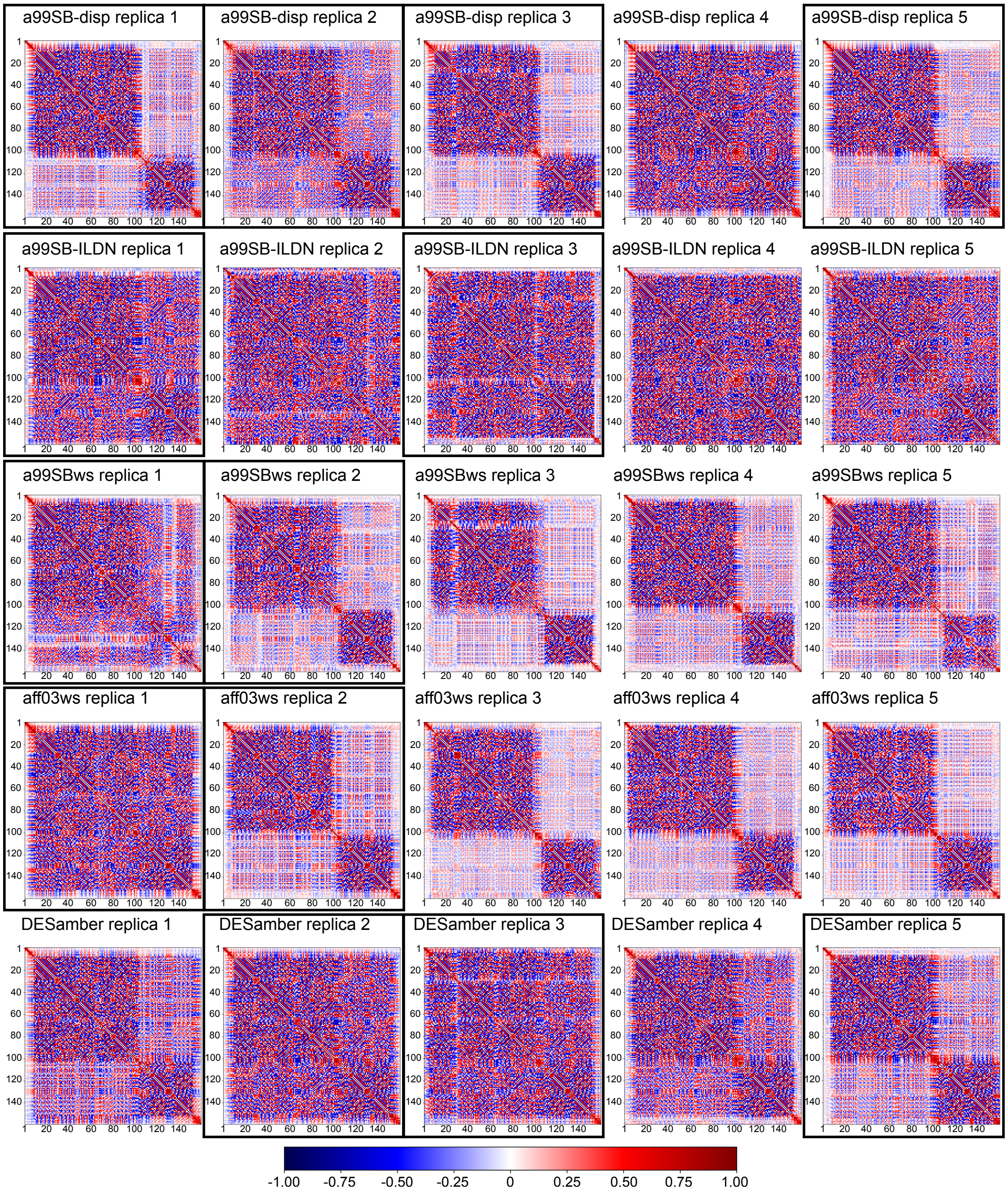

Figure S8: Protein backbone orientation correlation maps for vectors between C^{α} carbons of consecutive residues of all molecular dynamics (MD) simulation replicas for CDNF. QEBSS-selected replicas are highlighted with a black box.

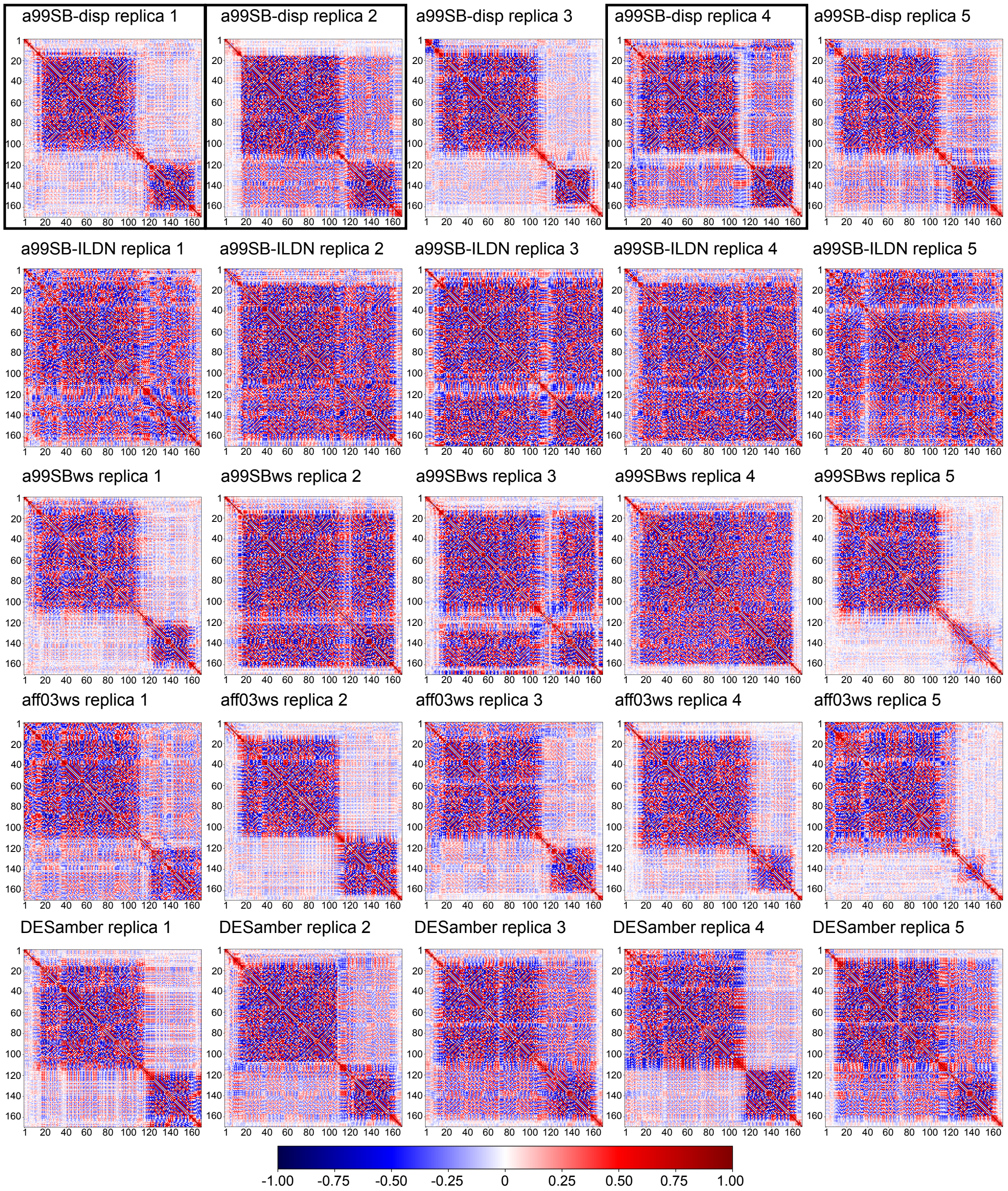

Figure S9: Protein backbone orientation correlation maps for vectors between C^{α} carbons of consecutive residues of all molecular dynamics (MD) simulation replicas for MANF. QEBSS-selected replicas are highlighted with a black box.

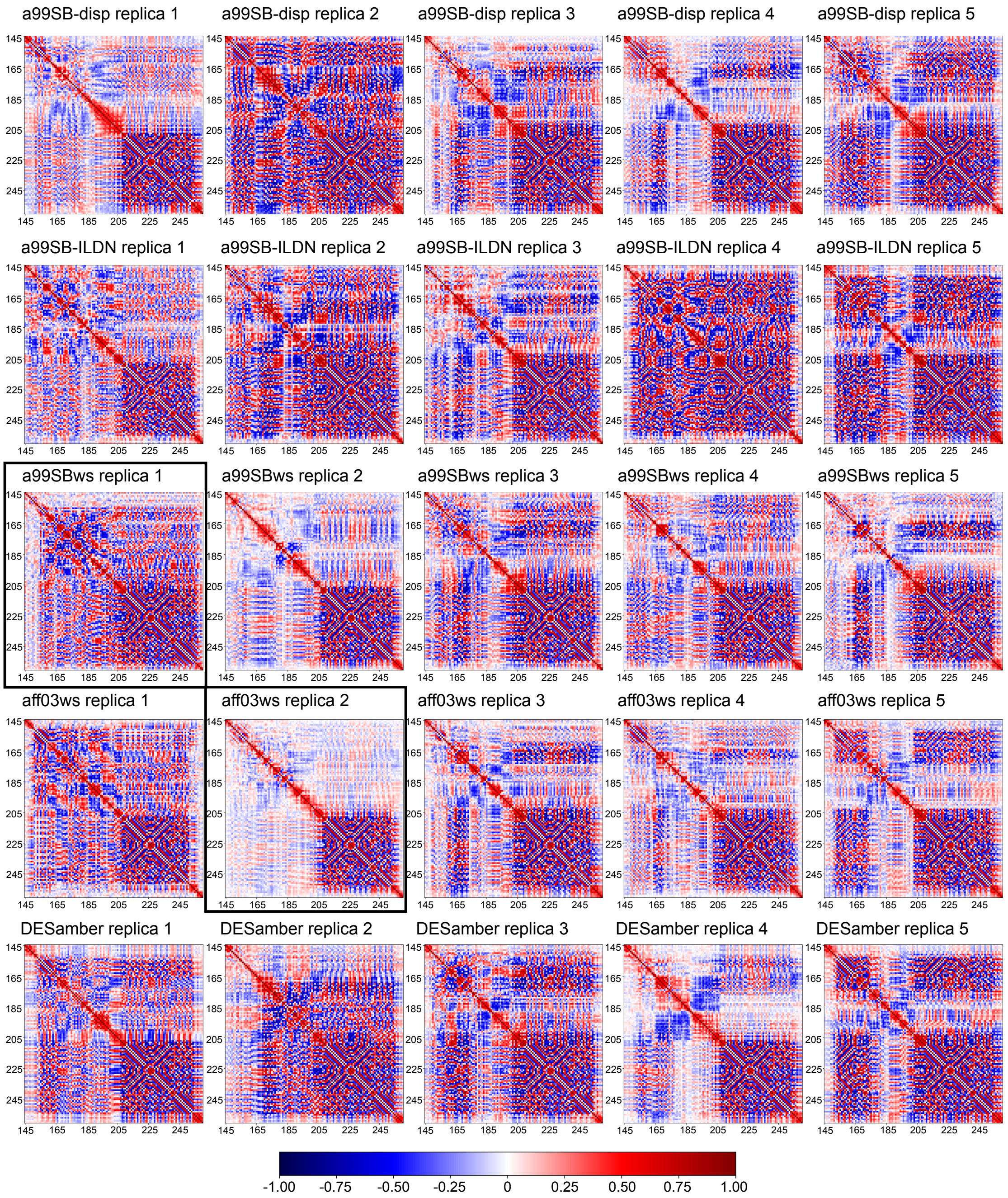

Figure S10: Protein backbone orientation correlation maps for vectors between C^{α} carbons of consecutive residues of all molecular dynamics (MD) simulation replicas for EN2. QEBSS-selected replicas are highlighted with a black box.

Comparison of spin relaxation times between simulations and experiments

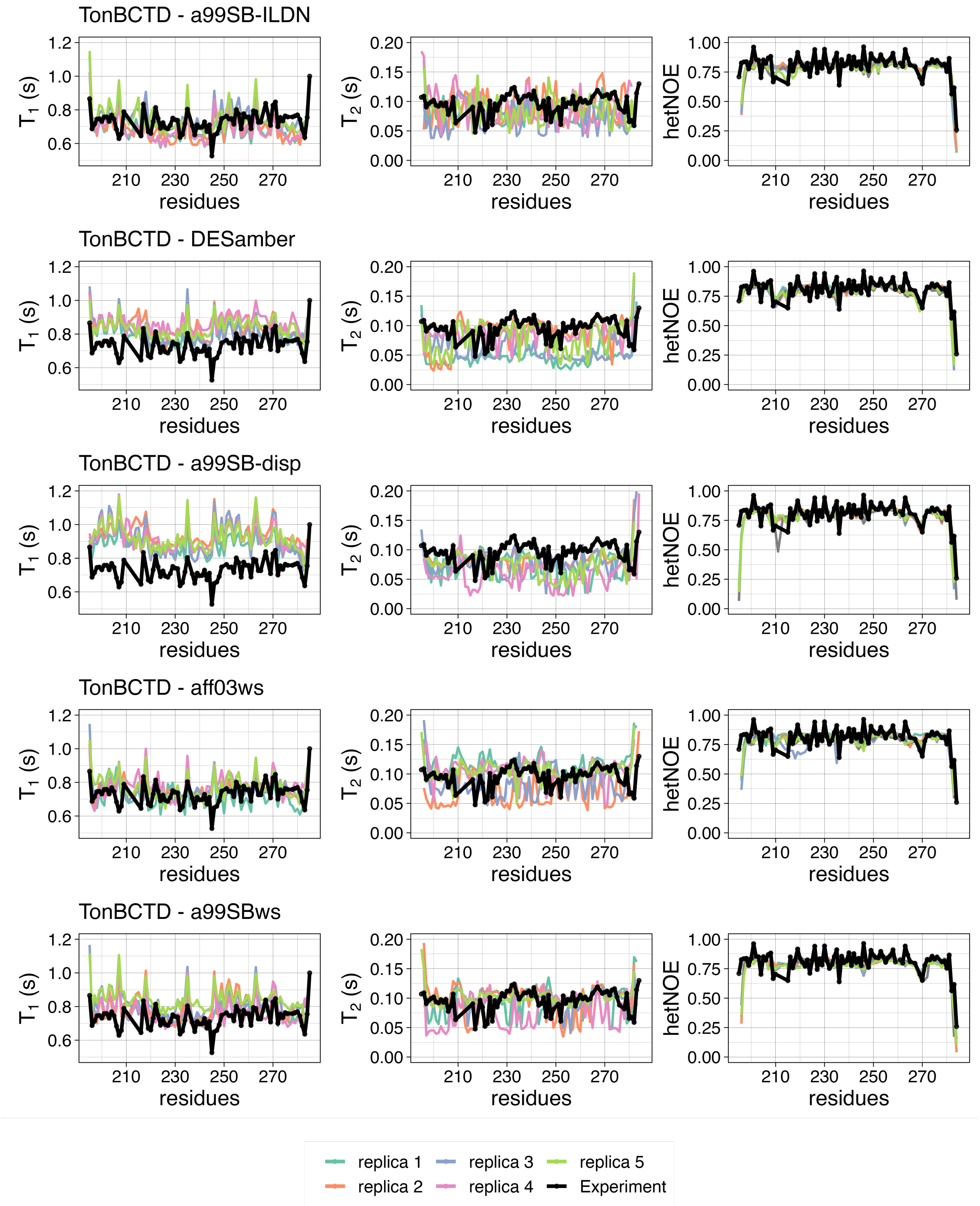

Figure S11: Calculated ^{15}N spin relaxation times, T_1 and T_2, and hetNOE values from each simulation replica with five different force fields compared to experimental spin relaxation data for TonBCTD[1]

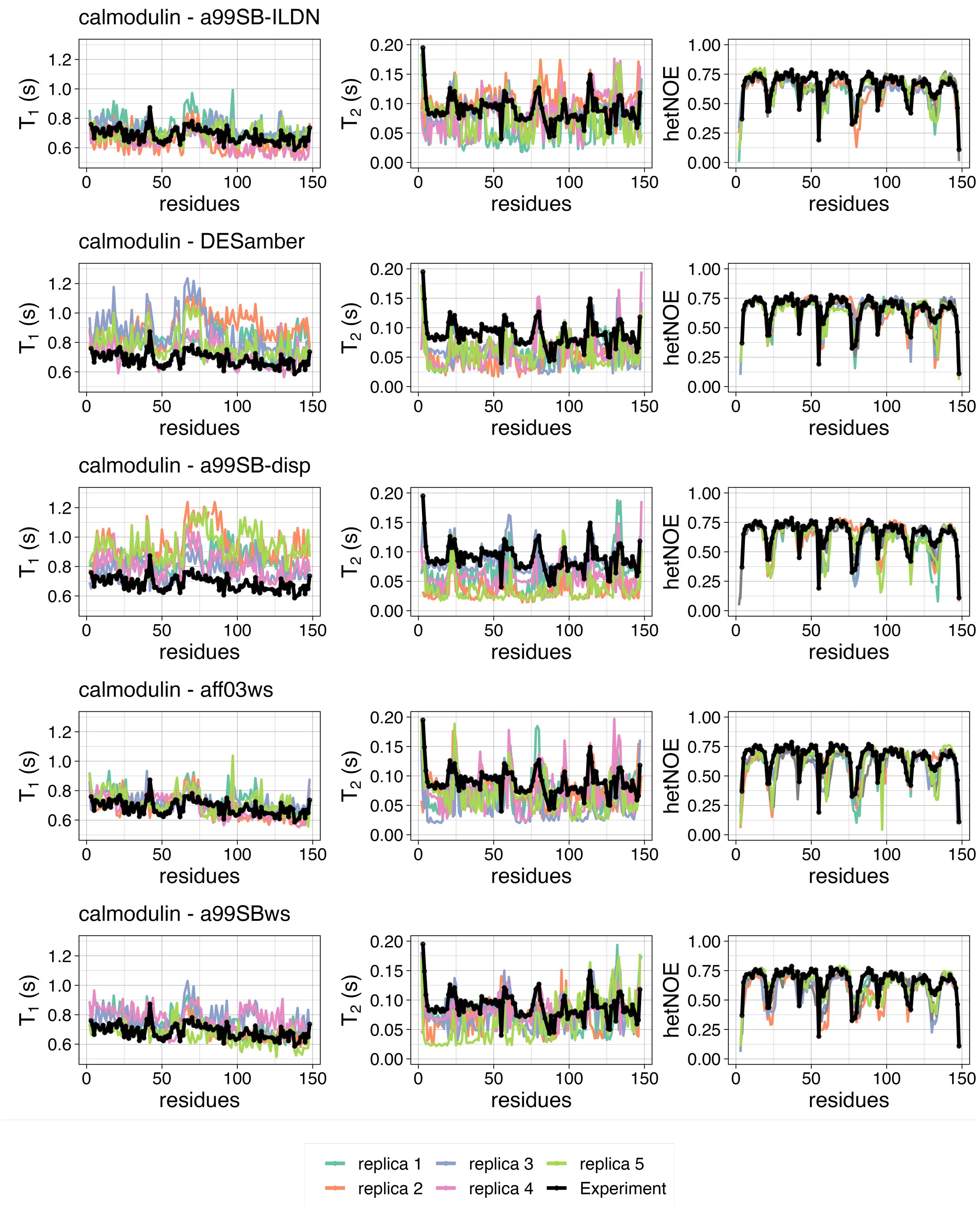

Figure S12: Calculated ^{15}N spin relaxation times, T_1 and T_2, and hetNOE values from each simulation replica with five different force fields compared to experimental spin relaxation data for calmodulin[2]

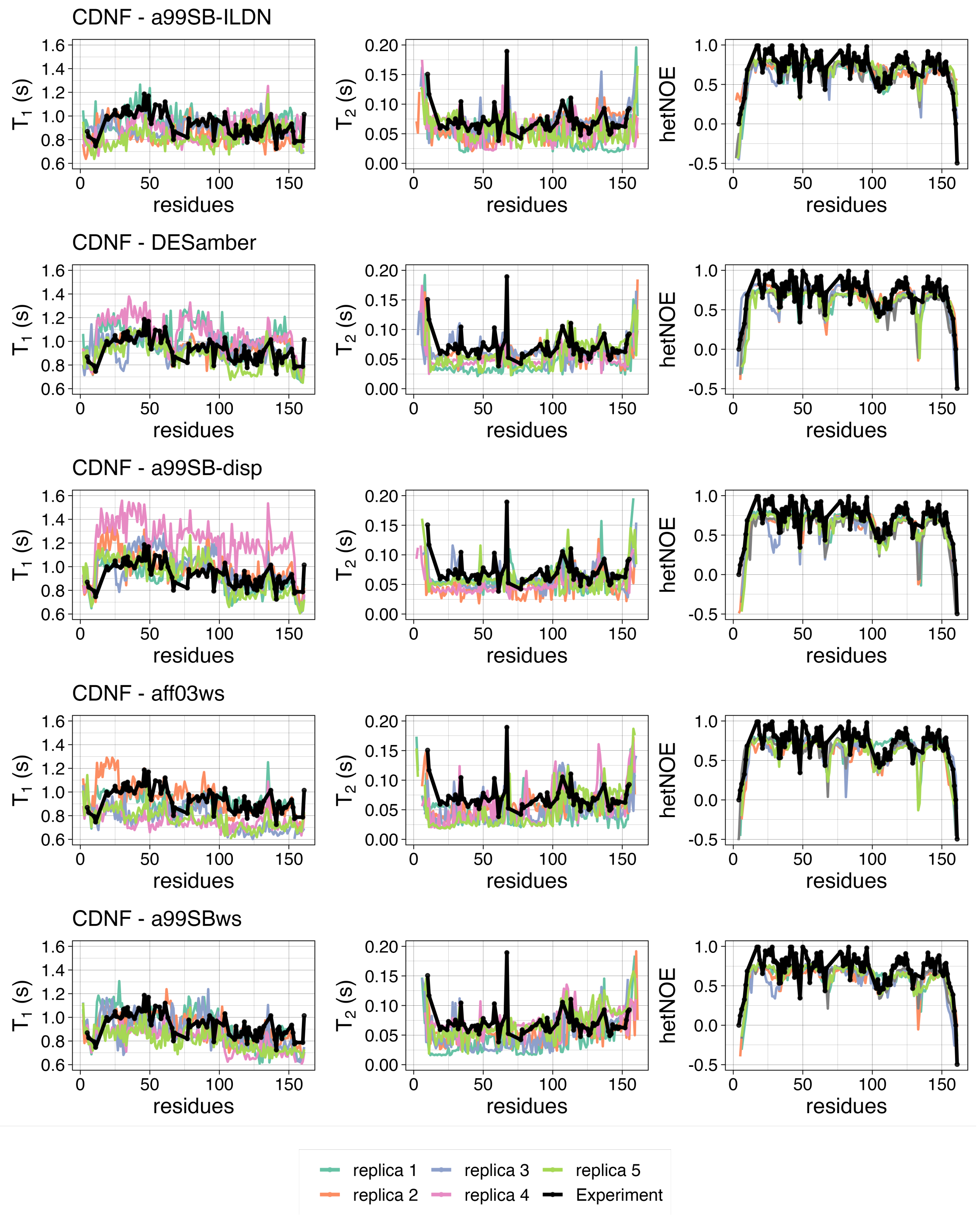

Figure S13: Calculated ^{15}N spin relaxation times, T_1 and T_2, and hetNOE values from each simulation replica with five different force fields compared to experimental spin relaxation data for CDNF[3]

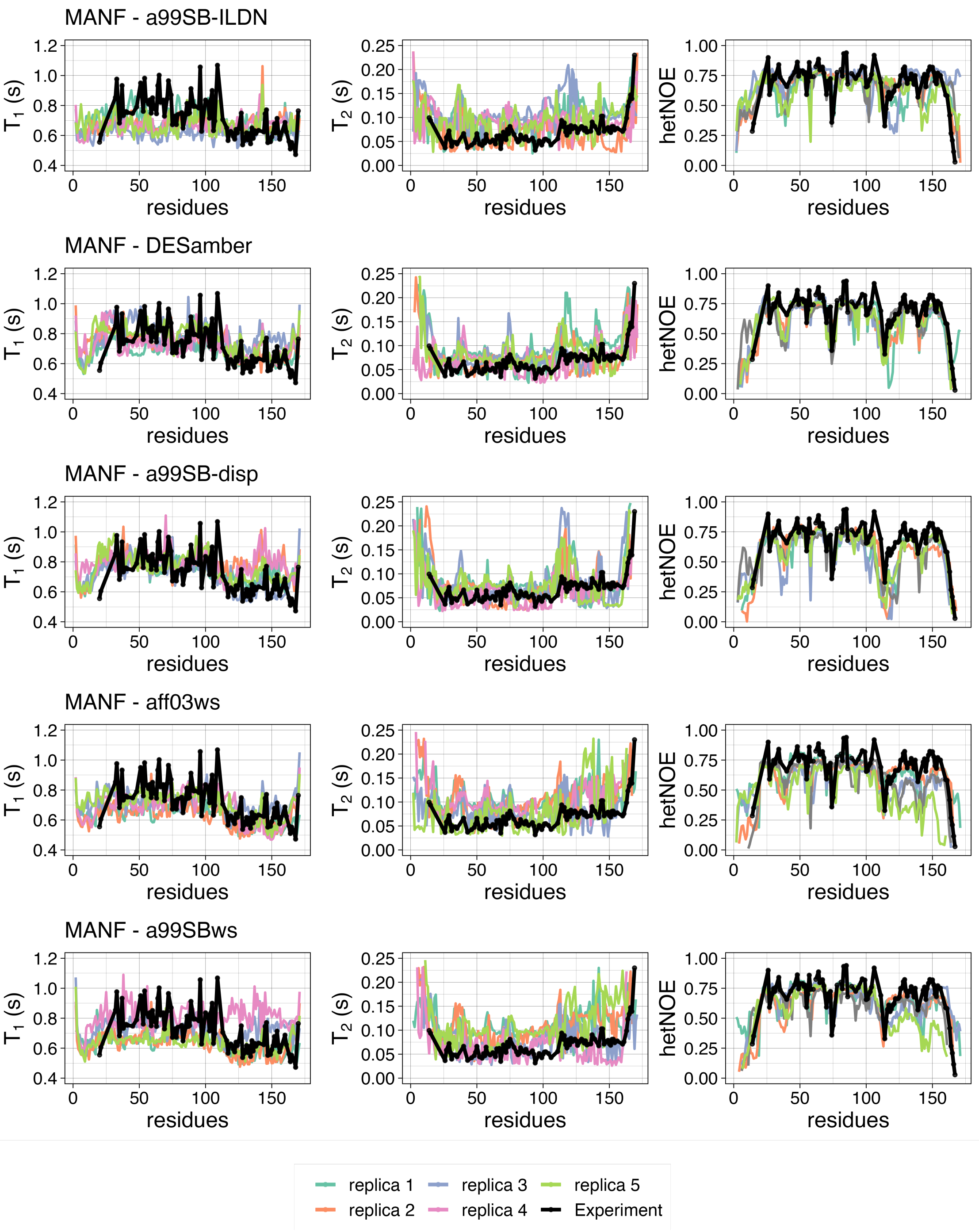

Figure S14: Calculated ^{15}N spin relaxation times, T_1 and T_2, and hetNOE values from each simulation replica with five different force fields compared to experimental spin relaxation data for MANF[4]

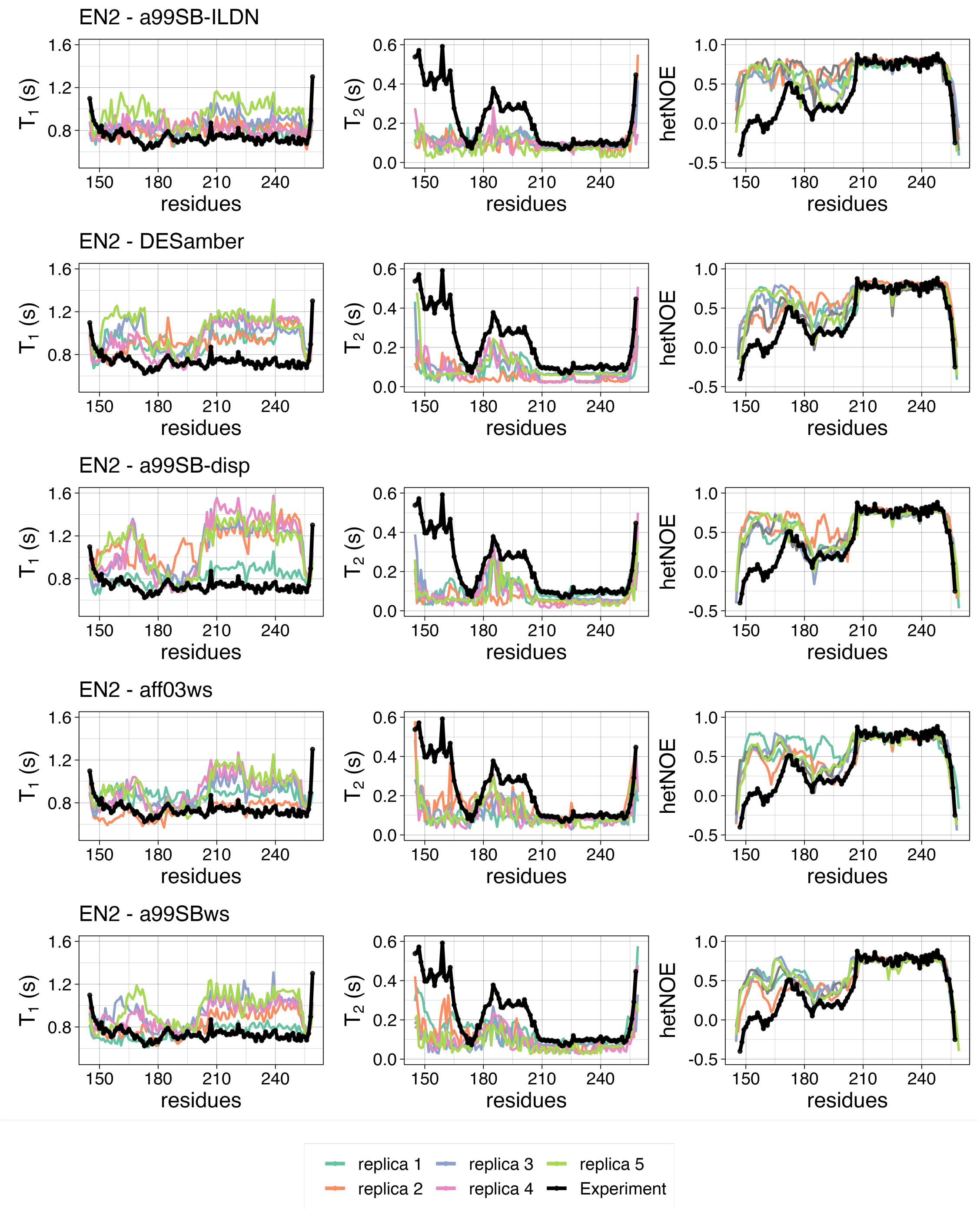

Figure S15: Calculated ^{15}N spin relaxation times, T_1 and T_2, and hetNOE values from each simulation replica with five different force fields compared to experimental spin relaxation data for EN2[5]

Comparison of SAXS intensities between simulations and experiments

Small angle x-ray scattering (SAXS) intensities calculated from the QEBSS ensembles are compared with the experimental data in the left column in Fig. S16. SAXS data from QEBSS ensembles are in excellent agreement with experiments for calmodulin and CDNF, and in good agreement or MANF. However, it should be noted that the experimental SAXS data for calmodulin[6] is from calcium-bound state while NMR data used in QEBSS ensemble is from calcium-free state.[2] Furthermore, NMR data and QEBSS ensemble for MANF are from mouse sequence with a 6xHis tag,[4] while SAXS data is from human sequence without the His tag. For CDNF, NMR[3] and SAXS data are from the same sequence and conditions.

Right column in Fig. S16 shows SAXS results from simulations with different conformational ensembles, spin relaxation times, and thus rankings in the QEBSS selection. The results indicate that many simulations predicting significantly different spin relaxation times (and thus ranking differently within the QEBSS protocol) exhibit relatively similar SAXS profiles. The exception was EN2, where the SAXS profile of the top ranked simulation with the extended conformational ensemble was clearly different than from bottom-ranked simulations with more compact conformations. However, experimental data for EN2 is not available for comparison. These results suggest that NMR spin relaxation times may be more sensitive to conformational ensembles of the studied proteins than SAXS data.

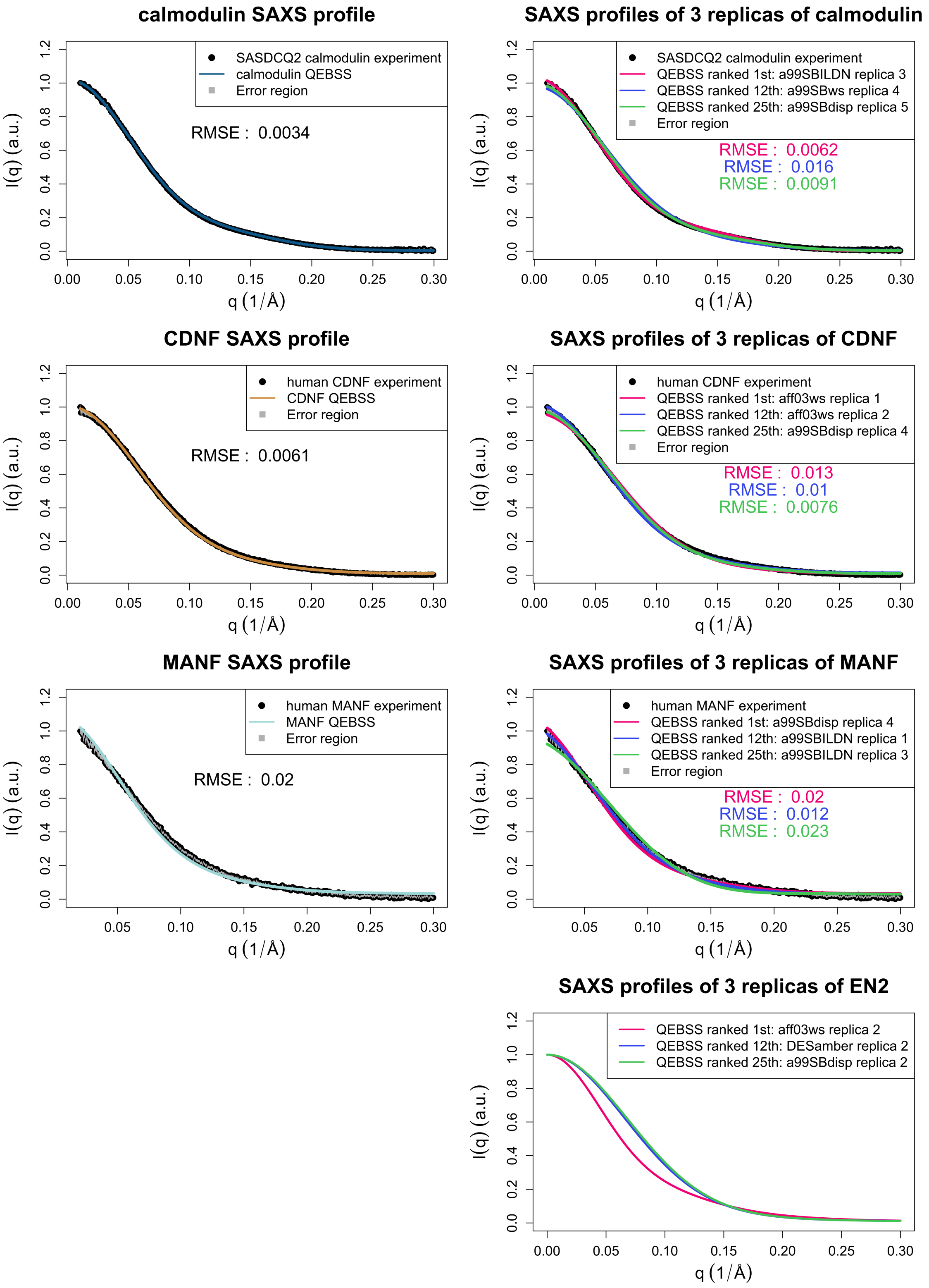

Figure S16: (*left*) SAXS intensities from QEBSS ensemble and experiments for calmodulin,[6] CDNF and MANF. (*right*) SAXS intensities calculated from three replicas ranked by QEBSS as 1st, 12th and 25th for calmodulin, CDNF, MANF and EN2.

Comparison of spin relaxation times from QEBSS ensembles to experimental data in different magnetic fields

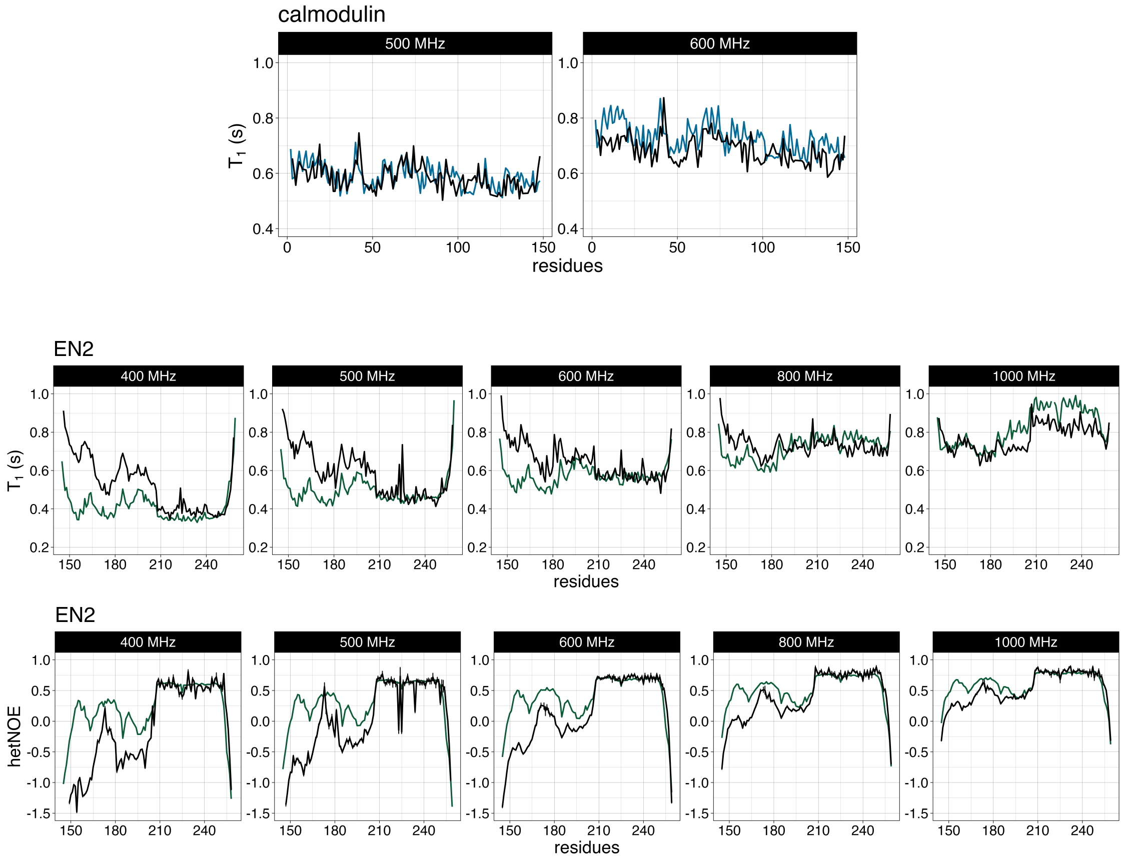

Figure S17: ^{15}N spin relaxation times, T_1 and hetNOE values from calmodulin (blue) and EN2 (green) calculated from QEBSS ensembles compared to experimental spin relaxation data (black) in multiple magnetic fields for calmodulin[2] and EN2[5]

Ranking of simulations against experimental spin relaxation times

Table S1: RMSDs and QEBSS comparison values for TonBCTD. RMSDs were calculated for ^{15}N spin relaxation times, T_1 and T_2, and hetNOE between simulations and experiments by averaging over residues. QEBSS comparison values were determined separately for T_1 and T_2, and hetNOE with respect to the simulation with the lowest RMSD for the given spin relaxation time. Replicas deviating less than 50 % from the simulation with the lowest RMSD (comparison number below 150%) for all spin relaxation experiments were selected for further analysis and are denoted by bold highlighting. Simulations are organized according to the sum of comparison numbers shown in the rightmost column.

Force field	Replica	T1 RMSD	T1 (%)	T2 RMSD	T2 (%)	hetNOE RMSD	hetNOE (%)	Sum (%)
**QEBSS**		**0.07**	**102.81**	**0.04**	**116.02**	**0.14**	**130.41**	**349.24**
**aff03ws**	**replica 2**	**0.07**	**100.00**	**0.04**	**113.24**	**0.14**	**126.44**	**339.68**
**aff03ws**	**replica 5**	**0.08**	**114.47**	**0.04**	**108.02**	**0.13**	**125.32**	**347.81**
**a99SB-ILDN**	**replica 4**	**0.09**	**121.41**	**0.04**	**117.34**	**0.12**	**115.43**	**354.18**
aff03ws	replica 3	0.08	104.96	0.05	129.43	0.16	150.35	384.74
**a99SB-ILDN**	**replica 5**	**0.08**	**111.78**	**0.05**	**146.41**	**0.14**	**128.64**	**386.84**
**aff03ws**	**replica 4**	**0.09**	**118.49**	**0.05**	**138.22**	**0.14**	**131.11**	**387.82**
**aff03ws**	**replica 1**	**0.09**	**120.41**	**0.05**	**144.79**	**0.14**	**132.77**	**397.98**
**a99SB-ILDN**	**replica 1**	**0.09**	**128.39**	**0.05**	**147.12**	**0.13**	**125.11**	**400.62**
a99SB-ILDN	replica 3	0.08	113.04	0.06	164.79	0.15	135.86	413.69
**a99SBws**	**replica 1**	**0.09**	**128.84**	**0.05**	**146.66**	**0.15**	**138.67**	**414.17**
DESamber	replica 2	0.13	173.71	0.04	116.73	0.15	136.26	426.71
a99SBws	replica 5	0.14	191.66	0.04	100.00	0.15	136.27	427.93
**a99SBws**	**replica 4**	**0.10**	**143.06**	**0.05**	**147.80**	**0.15**	**139.72**	**430.58**
DESamber	replica 5	0.12	170.82	0.05	131.25	0.14	129.58	431.66
a99SB-disp	replica 1	0.16	220.56	0.04	110.40	0.11	101.42	432.38
DESamber	replica 3	0.12	159.51	0.06	154.28	0.15	137.68	451.47
DESamber	replica 4	0.15	207.21	0.04	117.61	0.14	127.00	451.82
a99SBws	replica 3	0.13	176.40	0.05	126.19	0.16	149.28	451.86
a99SB-ILDN	replica 2	0.09	129.75	0.07	191.03	0.15	140.44	461.22
a99SBws	replica 2	0.12	160.92	0.06	160.21	0.16	146.84	467.97
DESamber	replica 1	0.11	144.77	0.07	190.37	0.14	134.72	469.87
a99SB-disp	replica 4	0.21	285.32	0.05	127.55	0.11	100.00	512.87
a99SB-disp	replica 5	0.21	294.67	0.05	125.75	0.12	111.98	532.41
a99SB-disp	replica 3	0.22	298.66	0.05	131.89	0.12	108.45	539.00
a99SB-disp	replica 2	0.23	309.15	0.04	117.47	0.12	114.14	540.76

Table S2: RMSDs and QEBSS comparison values for calmodulin. RMSDs were calculated for T_1 and T_2, and hetNOE between simulations and experiments by averaging over residues. QEBSS comparison values were determined separately for T_1 and T_2, and hetNOE with respect to the simulation with the lowest RMSD for the given spin relaxation time. Replicas deviating less than 50 % from the simulation with the lowest RMSD (comparison number below 150%) for all spin relaxation experiments were selected for further analysis and are denoted by bold highlighting. Simulations are organized according to the sum of comparison numbers shown in the rightmost column.

Force field	Replica	T1 RMSD	T1 (%)	T2 RMSD	T2 (%)	hetNOE RMSD	hetNOE (%)	Sum (%)
**QEBSS**		**0.06**	**108.15**	**0.03**	**118.29**	**0.09**	**127.33**	**353.77**
**a99SB-ILDN**	**replica 3**	**0.07**	**113.79**	**0.03**	**131.48**	**0.09**	**137.18**	**382.45**
DESamber	replica 4	0.07	112.80	0.04	169.53	0.07	106.14	388.46
**a99SBws**	**replica 1**	**0.08**	**142.50**	**0.03**	**133.95**	**0.08**	**114.75**	**391.21**
a99SB-ILDN	replica 4	0.07	122.62	0.04	169.35	0.08	116.89	408.85
a99SB-disp	replica 3	0.12	201.19	0.02	100.00	0.08	125.16	426.35
a99SB-ILDN	replica 2	0.08	138.67	0.03	153.69	0.10	142.09	434.45
a99SB-ILDN	replica 5	0.06	103.42	0.04	199.90	0.09	134.18	437.50
a99SBws	replica 2	0.06	100.00	0.04	172.45	0.13	188.27	460.72
a99SBws	replica 5	0.07	115.62	0.05	228.86	0.09	132.49	476.97
aff03ws	replica 2	0.07	113.39	0.04	183.94	0.12	179.69	477.02
aff03ws	replica 4	0.06	111.88	0.04	175.53	0.13	192.43	479.84
a99SBws	replica 4	0.12	209.05	0.03	129.00	0.10	144.38	482.43
a99SBws	replica 3	0.10	170.51	0.03	158.97	0.11	157.96	487.44
aff03ws	replica 1	0.08	134.55	0.04	183.10	0.12	175.48	493.14
aff03ws	replica 3	0.07	117.36	0.05	226.59	0.11	162.44	506.39
a99SB-ILDN	replica 1	0.09	154.56	0.05	213.24	0.09	141.13	508.93
DESamber	replica 1	0.14	238.56	0.04	176.81	0.07	100.00	515.37
aff03ws	replica 5	0.08	136.79	0.04	201.31	0.12	185.50	523.60
DESamber	replica 5	0.12	208.40	0.04	205.65	0.08	122.48	536.54
a99SB-disp	replica 4	0.14	240.02	0.04	197.09	0.07	103.83	540.93
a99SB-disp	replica 1	0.18	304.76	0.04	192.57	0.12	184.52	681.84
DESamber	replica 3	0.20	345.01	0.04	205.65	0.09	134.21	684.87
DESamber	replica 2	0.24	407.81	0.05	235.36	0.10	154.06	797.23
a99SB-disp	replica 2	0.28	487.64	0.06	289.73	0.11	159.48	936.86
a99SB-disp	replica 5	0.28	478.18	0.06	279.72	0.12	184.63	942.53

Table S3: RMSDs and QEBSS comparison values for CDNF. RMSDs were calculated for ^{15}N spin relaxation times, T_1 and T_2, and hetNOE between simulations and experiments by averaging over residues. QEBSS comparison values were determined separately for T_1 and T_2, and hetNOE with respect to the simulation with the lowest RMSD for the given spin relaxation time. Replicas deviating less than 50 % from the simulation with the lowest RMSD (comparison number below 150%) for all spin relaxation experiments were selected for further analysis and are denoted by bold highlighting. Simulations are organized according to the sum of comparison numbers shown in the rightmost column.

**Force Field**	**Replica**	**T1 RMSD**	**T1 (%)**	**T2 RMSD**	**T2 (%)**	**hetNOE RMSD**	**hetNOE (%)**	**Sum (%)**
**QEBSS**		**0.08**	**79.72**	**0.22**	**115.57**	**0.17**	**123.48**	**318.77**
**aff03ws**	**replica 1**	**0.09**	**100.00**	**0.22**	**116.03**	**0.17**	**119.95**	**335.97**
**DESamber**	**replica 2**	**0.10**	**107.82**	**0.24**	**123.31**	**0.15**	**106.82**	**337.95**
**DESamber**	**replica 3**	**0.12**	**128.55**	**0.20**	**104.20**	**0.16**	**111.56**	**344.32**
**a99SB-disp**	**replica 1**	**0.11**	**114.54**	**0.19**	**100.00**	**0.18**	**130.74**	**345.29**
**a99SB-disp**	**replica 2**	**0.13**	**135.10**	**0.20**	**104.03**	**0.16**	**115.36**	**354.48**
**a99SB-ILDN**	**replica 3**	**0.13**	**131.98**	**0.23**	**122.38**	**0.15**	**104.85**	**359.21**
**a99SBws**	**replica 2**	**0.10**	**105.95**	**0.25**	**128.74**	**0.18**	**125.80**	**360.49**
**DESamber**	**replica 5**	**0.11**	**117.47**	**0.24**	**125.82**	**0.18**	**126.99**	**370.28**
**a99SB-ILDN**	**replica 1**	**0.13**	**132.61**	**0.25**	**129.22**	**0.16**	**115.07**	**376.89**
**a99SB-disp**	**replica 3**	**0.12**	**129.52**	**0.20**	**107.06**	**0.20**	**141.96**	**378.55**
**a99SBws**	**replica 1**	**0.12**	**126.34**	**0.22**	**117.40**	**0.19**	**138.17**	**381.91**
**aff03ws**	**replica 2**	**0.11**	**115.29**	**0.23**	**121.84**	**0.21**	**148.64**	**385.76**
a99SB-disp	replica 5	0.13	136.35	0.22	116.26	0.20	142.48	395.09
a99SBws	replica 4	0.17	176.47	0.21	111.28	0.16	111.69	399.44
a99SB-ILDN	replica 4	0.16	167.98	0.25	129.73	0.14	102.78	400.49
a99SB-ILDN	replica 5	0.17	181.43	0.24	125.26	0.14	100.00	406.70
a99SB-ILDN	replica 2	0.15	155.91	0.25	131.66	0.17	120.58	408.15
a99SBws	replica 3	0.15	155.84	0.21	108.85	0.20	144.07	408.76
a99SBws	replica 5	0.16	171.01	0.23	119.57	0.17	120.77	411.34
DESamber	replica 1	0.16	165.17	0.23	119.58	0.19	137.56	422.31
aff03ws	replica 4	0.21	221.49	0.20	103.74	0.15	105.10	430.33
DESamber	replica 4	0.18	184.68	0.25	128.81	0.17	120.53	434.02
aff03ws	replica 5	0.19	203.63	0.20	106.26	0.18	127.16	437.06
aff03ws	replica 3	0.20	214.11	0.22	117.96	0.19	132.08	464.15
a99SB-disp	replica 4	0.33	342.71	0.19	101.62	0.19	137.00	581.33

Table S4: RMSDs and QEBSS comparison values for MANF. RMSDs were calculated for ^{15}N spin relaxation times, T_1 and T_2, and hetNOE between simulations and experiments by averaging over residues. QEBSS comparison values were determined separately for T_1 and T_2, and hetNOE with respect to the simulation with the lowest RMSD for the given spin relaxation time. Replicas deviating less than 50 % from the simulation with the lowest RMSD (comparison number below 150%) for all spin relaxation experiments were selected for further analysis and are denoted by bold highlighting. Simulations are organized according to the sum of comparison numbers shown in the rightmost column.

Force field	Replica	T1 RMSD	T1 (%)	T2 RMSD	T2 (%)	hetNOE RMSD	hetNOE (%)	Sum (%)
**QEBSS**		**0.13**	**117.29**	**0.03**	**102.49**	**0.15**	**147.14**	**366.92**
**a99SB-disp**	**replica 4**	**0.14**	**128.44**	**0.03**	**100.00**	**0.13**	**125.89**	**354.33**
**a99SB-disp**	**replica 1**	**0.12**	**107.35**	**0.04**	**145.41**	**0.12**	**119.50**	**372.26**
**a99SB-disp**	**replica 2**	**0.14**	**127.51**	**0.03**	**123.35**	**0.13**	**123.58**	**374.45**
DESamber	replica 5	0.12	103.09	0.04	162.28	0.12	116.18	381.55
DESamber	replica 2	0.11	101.84	0.05	190.09	0.10	100.00	391.94
a99SB-disp	replica 5	0.12	104.29	0.05	183.35	0.12	110.75	398.39
DESamber	replica 4	0.12	110.02	0.05	194.95	0.10	100.93	405.91
a99SB-ILDN	replica 2	0.14	123.11	0.05	169.49	0.13	122.65	415.26
DESamber	replica 3	0.13	117.85	0.05	190.15	0.12	117.12	425.12
a99SBws	replica 2	0.15	131.11	0.05	196.78	0.11	102.27	430.16
aff03ws	replica 2	0.15	131.11	0.05	196.78	0.11	102.27	430.16
a99SB-ILDN	replica 1	0.12	110.55	0.05	184.77	0.16	149.62	444.94
a99SB-ILDN	replica 4	0.14	128.73	0.05	192.45	0.14	135.60	456.78
a99SBws	replica 3	0.13	115.60	0.05	199.39	0.16	152.34	467.34
a99SBws	replica 1	0.14	128.46	0.06	221.06	0.15	146.81	496.33
aff03ws	replica 1	0.14	128.46	0.06	221.06	0.15	146.81	496.33
a99SB-ILDN	replica 5	0.14	124.24	0.05	199.84	0.18	175.68	499.75
aff03ws	replica 4	0.13	119.12	0.06	234.48	0.15	146.66	500.26
DESamber	replica 1	0.12	106.56	0.06	229.84	0.17	166.70	503.10
aff03ws	replica 3	0.13	113.64	0.07	256.52	0.16	154.52	524.68
a99SB-disp	replica 3	0.12	107.62	0.07	242.61	0.18	176.31	526.55
a99SBws	replica 4	0.19	166.92	0.06	224.94	0.15	142.14	534.00
a99SBws	replica 5	0.15	132.70	0.07	256.44	0.18	169.64	558.77
aff03ws	replica 5	0.11	100.00	0.07	248.34	0.22	213.30	561.64
a99SB-ILDN	replica 3	0.17	148.33	0.07	254.43	0.21	202.52	605.29

Table S5: RMSDs and QEBSS comparison values for EN2. RMSDs were calculated for ^{15}N spin relaxation times, T_1 and T_2, and hetNOE between simulations and experiments by averaging over residues. QEBSS comparison values were determined separately for T_1 and T_2, and hetNOE with respect to the simulation with the lowest RMSD for the given spin relaxation time. Replicas deviating less than 50 % from the simulation with the lowest RMSD (comparison number below 150%) for all spin relaxation experiments were selected for further analysis and are denoted by bold highlighting. Simulations are organized according to the sum of comparison numbers shown in the rightmost column.

Force field	Replica	T1 RMSD	T1 (%)	T2 RMSD	T2 (%)	hetNOE RMSD	hetNOE (%)	Sum (%)
**QEBSS**		**0.08**	**93.15**	**0.12**	**91.97**	**0.25**	**126.36**	**311.48**
**aff03ws**	**replica 2**	**0.09**	**100.00**	**0.14**	**106.63**	**0.24**	**119.47**	**326.11**
**a99SBws**	**replica 1**	**0.09**	**107.07**	**0.13**	**100.00**	**0.27**	**133.63**	**340.71**
a99SBws	replica 2	0.16	183.41	0.15	110.27	0.20	100.00	393.68
a99SB-ILDN	replica 1	0.10	115.84	0.17	126.35	0.38	190.57	432.76
a99SB-disp	replica 1	0.13	152.56	0.18	137.72	0.29	143.91	434.20
a99SB-ILDN	replica 2	0.13	143.80	0.18	137.36	0.41	203.00	484.15
a99SB-ILDN	replica 4	0.12	141.99	0.19	142.80	0.41	203.73	488.53
aff03ws	replica 3	0.20	230.23	0.16	118.77	0.28	141.57	490.57
a99SB-ILDN	replica 3	0.17	200.37	0.18	131.60	0.35	174.47	506.44
aff03ws	replica 1	0.17	196.63	0.19	141.90	0.37	187.26	525.78
a99SBws	replica 4	0.22	255.38	0.18	136.23	0.28	139.67	531.28
DESamber	replica 2	0.23	260.02	0.20	151.14	0.27	136.49	547.65
aff03ws	replica 5	0.25	291.12	0.16	122.15	0.27	134.47	547.74
aff03ws	replica 4	0.24	278.67	0.19	141.72	0.29	147.18	567.57
DESamber	replica 4	0.28	324.45	0.18	138.71	0.21	106.20	569.36
DESamber	replica 1	0.23	265.05	0.21	155.68	0.31	152.77	573.50
a99SBws	replica 3	0.25	284.80	0.19	144.33	0.30	151.92	581.05
DESamber	replica 3	0.29	328.26	0.17	129.98	0.30	149.91	608.16
a99SB-ILDN	replica 5	0.27	304.79	0.19	142.62	0.34	169.45	616.86
a99SBws	replica 5	0.29	333.94	0.19	145.09	0.29	144.33	623.36
DESamber	replica 5	0.34	395.23	0.17	126.60	0.30	148.82	670.64
a99SB-disp	replica 3	0.41	472.55	0.19	144.10	0.27	136.52	753.17
a99SB-disp	replica 5	0.43	493.71	0.20	149.19	0.33	164.61	807.51
a99SB-disp	replica 4	0.47	534.64	0.19	145.46	0.27	136.70	816.81
a99SB-disp	replica 2	0.43	492.75	0.21	159.96	0.35	175.27	827.98

Principal component analysis

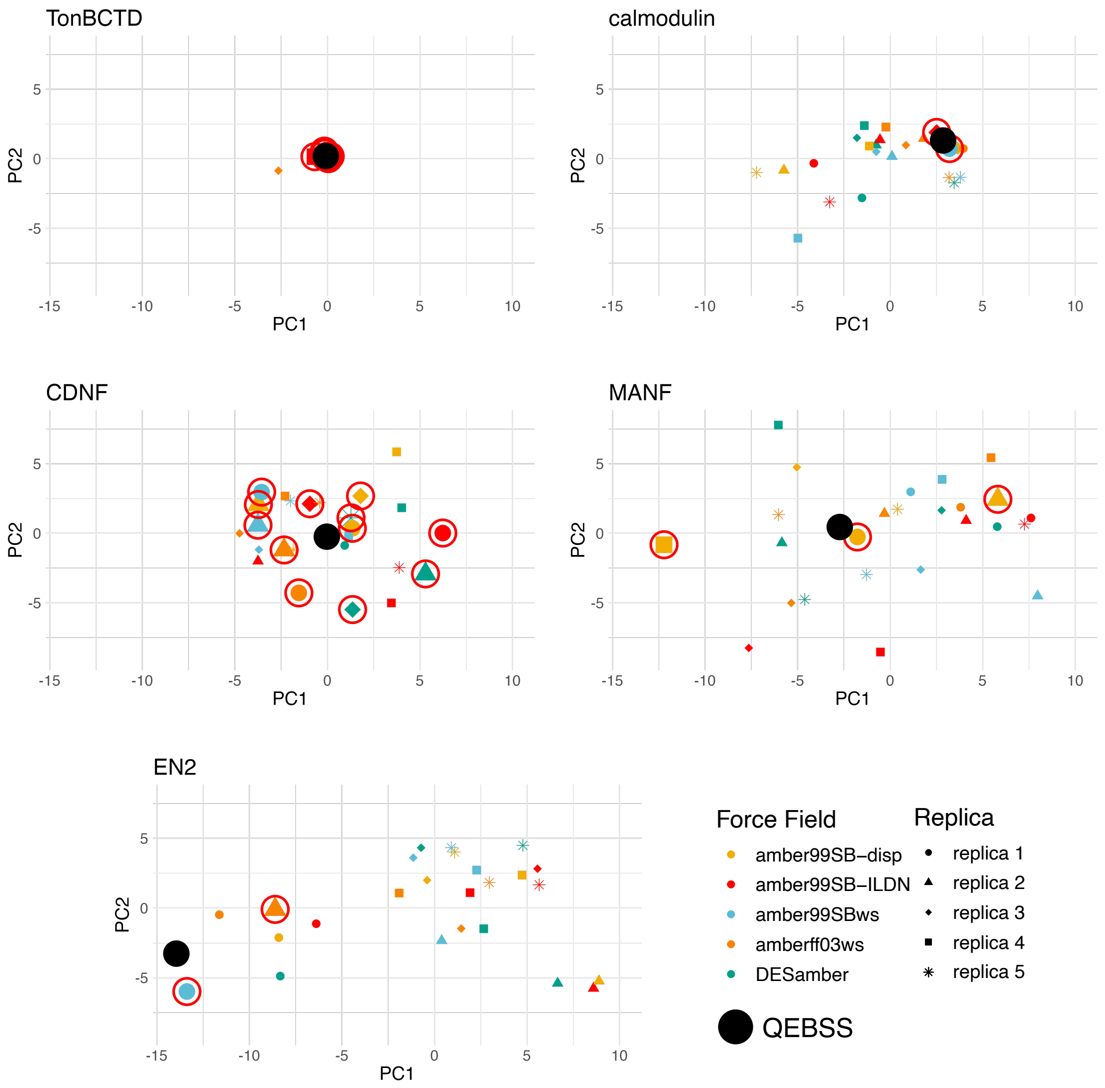

Figure S18: Principal component analysis of all 25 MD simulations and the QEBSS ensemble for TonBCTD, calmodulin, CDNF, MANF and EN2. Simulations selected for the QEBSS ensemble are highlighted with a red circle.

Analysis of starting structures

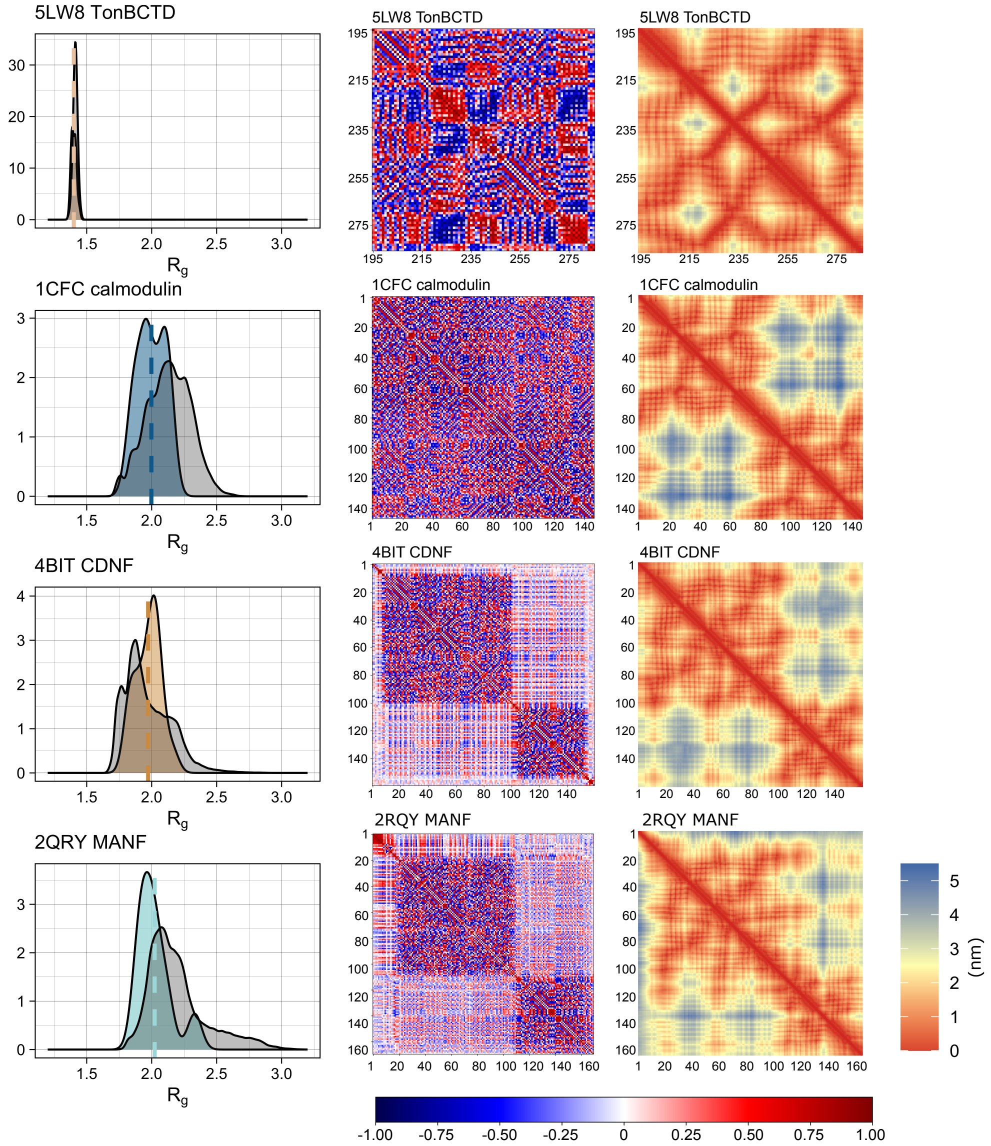

Figure S19: Radius of gyration (R_g in nm) distributions (*left*), protein backbone orientation correlation maps for vectors between C^α carbons of consecutive residues (*middle*), average minimum distance between residues maps (*right*) of the starting structure ensembles from the PDB databank for TonBCTD (PDB: 5LW8[7]), calmodulin (PDB: 1CFC[8]), CDNF (PDB: 4BIT[3]) and MANF (PDB: 2RQY[4]).

Dynamic landscapes of proteins

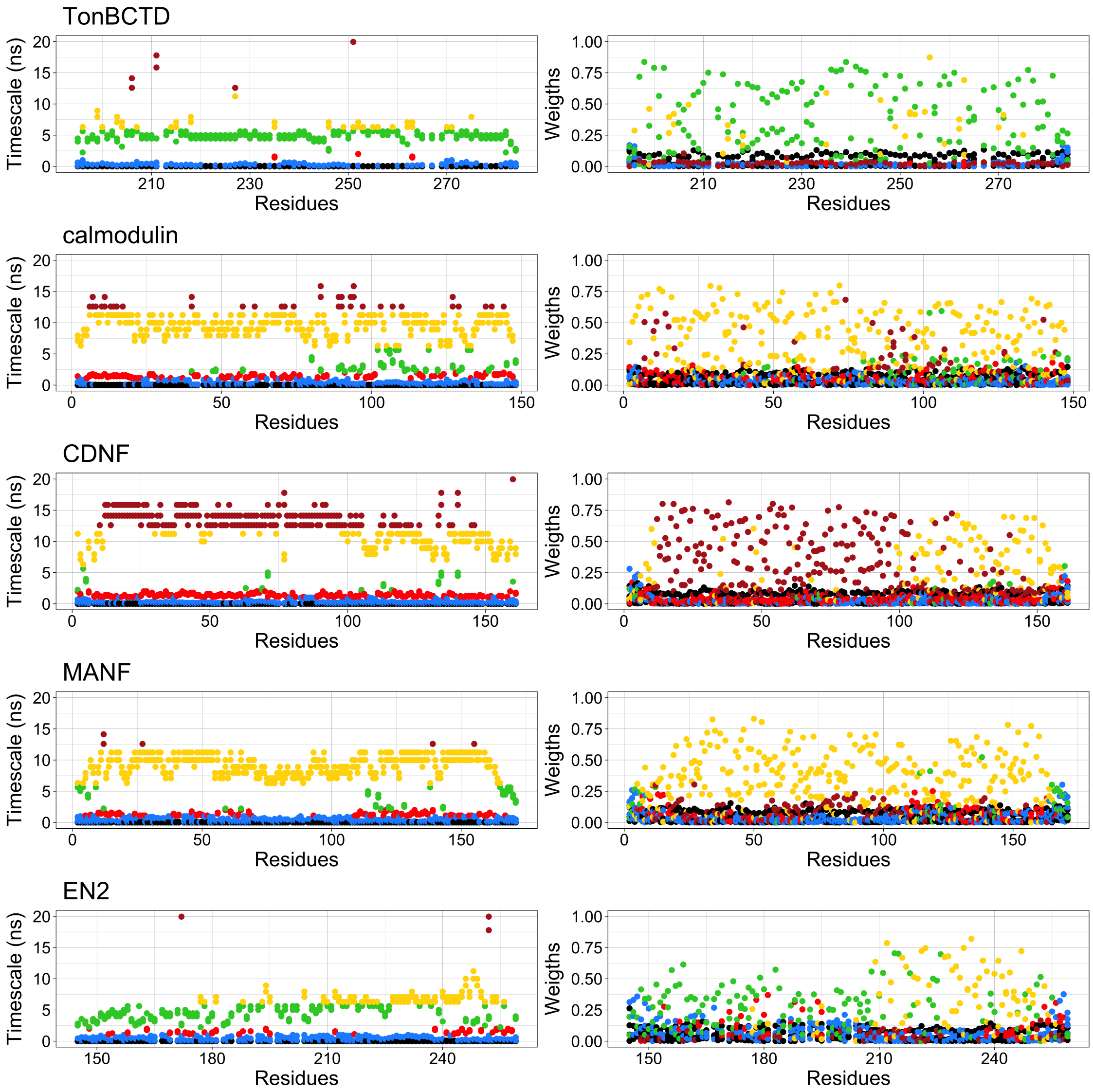

Figure S20: Timescales and weights of averaged correlation functions from QEBSS protocol selected simulations. Timescales $<$ 0.1 ns (black), 0.1 - 1 ns (blue), 1-2 ns (red), 2-6 ns (green), 6-12 ns (yellow), $<$ 12 ns (brown).

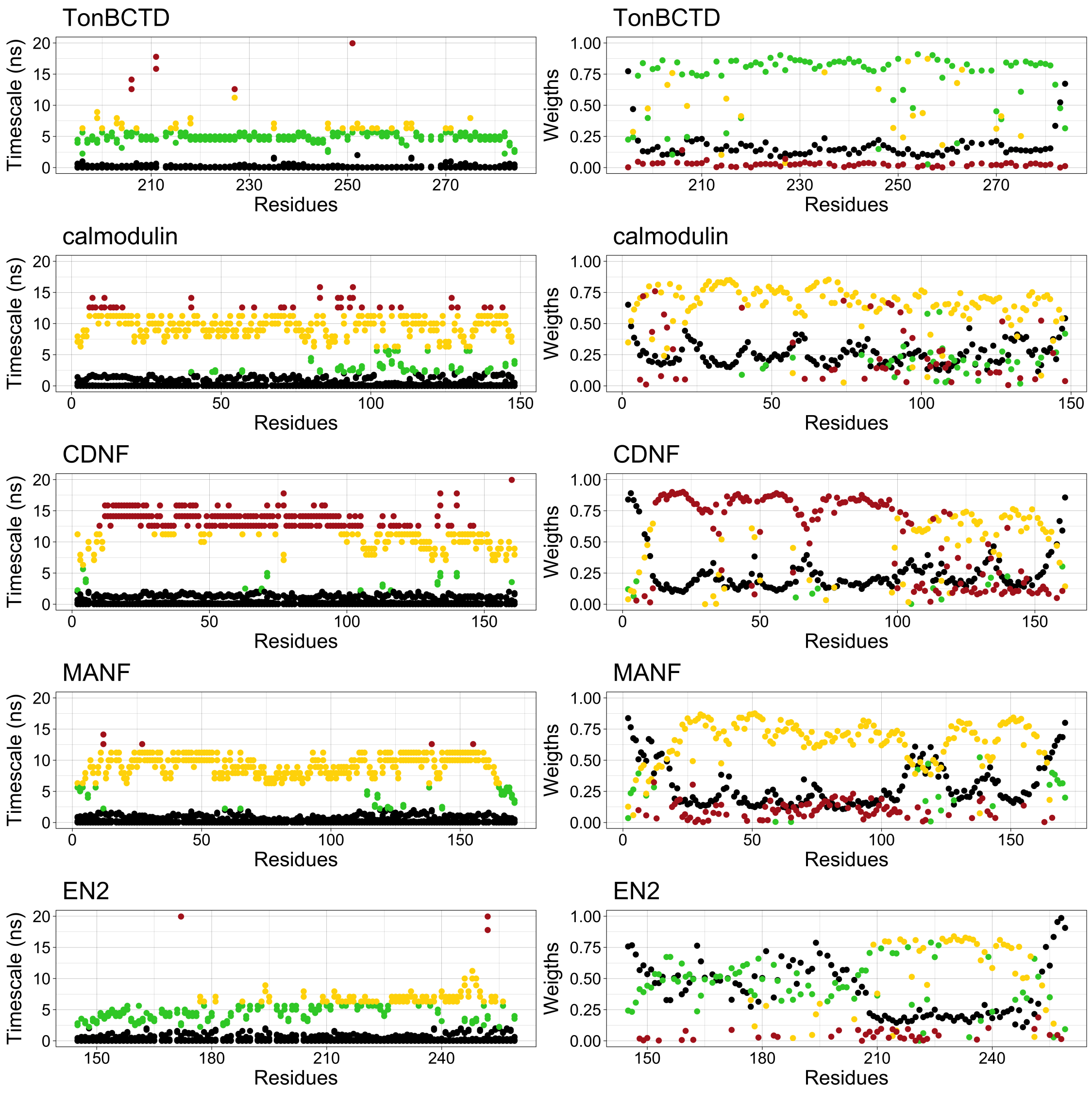

Figure S21: Timescales and weights of averaged correlation functions of QEBSS protocol selected simulations, where the weights for each interval has been summed. Timescales < 2 ns (black), 2-6 ns (green), 6-12 ns (yellow), < 12 ns (brown).

Simulation data availability

Table S6: All simulation data is available in Zenodo repositories.

Protein	Force Field	DOI
TonBCTD	a99SB-ILDN	https://doi.org/10.5281/zenodo.10952046
TonBCTD	DESamber	https://doi.org/10.5281/zenodo.11281995
TonBCTD	a99SB-disp	https://doi.org/10.5281/zenodo.11282835
TonBCTD	aff03ws	https://doi.org/10.5281/zenodo.11282871
TonBCTD	a99SBws	https://doi.org/10.5281/zenodo.11282884
calmodulin	a99SB-ILDN	https://doi.org/10.5281/zenodo.13235492
calmodulin	DESamber	https://doi.org/10.5281/zenodo.13235553
calmodulin	a99SB-disp	https://doi.org/10.5281/zenodo.13235667
calmodulin	aff03ws	https://doi.org/10.5281/zenodo.13235690
calmodulin	a99SBws	https://doi.org/10.5281/zenodo.13235704
CDNF	a99SB-ILDN	https://doi.org/10.5281/zenodo.13235725
CDNF	DESamber	https://doi.org/10.5281/zenodo.13235745
CDNF	a99SB-disp	https://doi.org/10.5281/zenodo.13235771
CDNF	aff03ws	https://doi.org/10.5281/zenodo.13236117
CDNF	a99SBws	https://doi.org/10.5281/zenodo.13236125
MANF	a99SB-ILDN	https://doi.org/10.5281/zenodo.13236162
MANF	DESamber	https://doi.org/10.5281/zenodo.13236168
MANF	a99SB-disp	https://doi.org/10.5281/zenodo.13236210
MANF	aff03ws	https://doi.org/10.5281/zenodo.13236216
MANF	a99SBws	https://doi.org/10.5281/zenodo.13236220
EN2	a99SB-ILDN	https://doi.org/10.5281/zenodo.13236228
EN2	DESamber	https://doi.org/10.5281/zenodo.13254426
EN2	a99SB-disp	https://doi.org/10.5281/zenodo.13254432
EN2	aff03ws	https://doi.org/10.5281/zenodo.13254436
EN2	a99SBws	https://doi.org/10.5281/zenodo.13254445

Supplementary References

(1) Oeemig, J. S.; Ollila, O. H. S.; Iwaï, H. NMR structure of the C-terminal domain of TonB protein from Pseudomonas aeruginosa. *PeerJ* **2018**, *6*, e5412.

(2) Tjandra, N.; Kuboniwa, H.; Ren, H.; Bax, A. Rotational Dynamics of Calcium-Free Calmodulin Studied by 15N-NMR Relaxation Measurements. *Eur. J. Biochem.* **1995**, *230*, 1014–1024.

(3) Latge, C.; Cabral, K. M. S.; de Oliveira, G. A. P.; Raymundo, D. P.; Freitas, J. A.; Johanson, L.; Romão, L. F.; Palhano, F. L.; Herrmann, T.; Almeida, M. S.; Foguel, D. The Solution Structure and Dynamics of Full-length Human Cerebral Dopamine Neurotrophic Factor and Its Neuroprotective Role against α-Synuclein Oligomers. *The Journal of biological chemistry* **2015**, *290*, 20527–40.

(4) Hoseki, J.; Sasakawa, H.; Yamaguchi, Y.; Maeda, M.; Kubota, H.; Kato, K.; Nagata, K. Solution structure and dynamics of mouse ARMET. *FEBS letters* **2010**, *584*, 1536–42.

(5) Khan, S. N.; Charlier, C.; Augustyniak, R.; Salvi, N.; Déjean, V.; Bodenhausen, G.; Lequin, O.; Pelupessy, P.; Ferrage, F. Distribution of Pico- and Nanosecond Motions in Disordered Proteins from Nuclear Spin Relaxation. *Biophysical Journal* **2015**, *109*, 988–999.

(6) Trewhella, J. et al. 2017 publication guidelines for structural modelling of small-angle scattering data from biomolecules in solution: an update. *Acta crystallographica. Section D, Structural biology* **2017**, *73*, 710—728.

(7) Ciragan, A.; Aranko, A. S.; Tascon, I.; Iwaï, H. Salt-inducible Protein Splicing in cis and trans by Inteins from Extremely Halophilic Archaea as a Novel Protein-Engineering Tool. *Journal of Molecular Biology* **2016**, *428*, 4573–4588.

(8) Kuboniwa, H.; Tjandra, N.; Grzesiek, S.; Ren, H.; Klee, C. B.; Bax, A. Solution structure of calcium-free calmodulin. *Nature structural biology* **1995**, *2*, 768–76.
